# Account of Diseases in an Eastern District of London, from the 20th of July to the 20th of August 1807

**Published:** 1807-09

**Authors:** 


*Account of Diseases in an Eastern District of London, from the 20th of July to the 20th of August* 1807.

| | |
|---|---|
| **ACUTE DISEASES.** | |
| Typhus - - - | 3 |
| Dysenteria - - | 2 |
| Cholera - - - | 15 |
| Pneumonia - - | 1 |
| Rheumatismus Acutus | 2 |
| **CHRONIC DISEASES.** | |
| Tussis - - - | 4 |
| Dyspnœa - - - | 7 |
| Hæmoptysis - - | 3 |
| Phthisis Pulmonalis - | 4 |
| Hepatitis Chronica - | 3 |
| Diarrhœa - - - | 7 |
| Gastrodynia - - | 5 |
| Menorrhagia - - | 4 |
| Chlorosis - - - | 3 |
| Dysuria - - - | 6 |
| Hysteria - - - | 5 |
| Herpes - - - | 4 |
| Scrophula - - | 3 |
| Cephalalgia - - | 4 |
| Rheumatismus Chronicus | 10 |
| **PUERPERAL DISEASES.** | |
| Menorrhagia Lochialis | 5 |
| Ephemera - - | 5 |
| Diarrhœa - - | 6 |
| Mastodynia - - | 5 |
| **INFANTILE DISEASES.** | |
| Rubeola - - - | 1 |
| Diarrhœa - - | 7 |
| Aphthæ - - - | 3 |
| Ophthalmia Purulenta | 2 |

Amongst the diseases which at present engage the attention of practitioners, cholera makes a distinguished figure. This has been divided by Nosologists into different species, in all of which a very large discharge of bilious and acrid matter from the bowels constitutes a principal symptom. These discharges frequently go on at the same time; and the quantity evacuated is truly surprising to those who are not acquainted with the nature of the disease. In some cases an uneasiness in the stomach and bowels is felt for some days previous to the principal attack; but in others this approaches more suddenly. Nausea accompanied by pain in the stomach, distention of the belly, and a considerable degree of flatulence, are observed. To these symptoms succeed violent vomiting and purging. What is first thrown up consists chiefly of food that has remained in the stomach in an undigested state; and the patient feeling some immediate relief, supposes that he shall soon be pesfectly well; but this is soon succeeded by a discharge of a very different appearance: bilious matter mixt with mucus is thrown up, and there are very considerable evacuations from the intestines of a similar appearance. Spasmodic contractions of the muscles of the legs generally attend this complaint. In one of the instances referred to in the list, these were transferred to the abdomen, and formed a very troublesome symptom.

The general indications of cure in this disease are, plentiful dilution of the offensive matter in the stomach and intestines, and the occasional use of opiates to correct violent action and alleviate pain.

